# Immobilization of cellulase on a silica gel substrate modified using a 3-APTES self-assembled monolayer

**DOI:** 10.1186/s40064-016-1682-y

**Published:** 2016-01-20

**Authors:** Dezhi Zhang, Hisham E. Hegab, Yuri Lvov, L. Dale Snow, James Palmer

**Affiliations:** 1 Chemical Engineering, Louisiana Tech University, 600 W. Arizona, Ruston, LA USA; 2 Mechanical Engineering, Louisiana Tech University, 600 W. Arizona, Ruston, LA USA; 3 Chemistry, Louisiana Tech University, 600 W. Arizona, Ruston, LA USA

**Keywords:** Cellulase, (3-Aminopropyl) triethoxy-silane, Glutaraldehyde, Glucose, Silica gel

## Abstract

Cellulase was immobilized onto silica gel surfaces pretreated with (3-aminopropyl) triethoxy-silane (3-APTES), and glutaraldehyde (GA) was used as a cross-linker. A carboxymethyl cellulose sodium salt (CMC) solution was used for activity experiments. Protein assay was performed to determine the mass immobilized and compare with free enzyme. Cellulase was successfully demonstrated to be immobilized on the modified silica gel surface, and no detectable amount of enzyme was stripped off during the hydrolysis of the CMC solution. The specific activity of the immobilized cellulase is 7 ± 2 % compared to the similar amount of free cellulase. Significant activity over multiple reuses was observed. The seventh batch achieved 82 % activity of the initial batch, and the fifteenth batch retained 31 %. It was observed that the immobilized cellulase retained 48 % of its initial activity after 4 days, and 22 % even after 14 days.

## Background

Bioethanol, biomethanol, biodiesel, etc. known as biofuels are an alternative energy source that will likely become more prevalent in the future (Nigam and Singh 2011). In the past, bioethanol has been made from plants that produced high amounts of natural sugar—such as sugar cane or corn. Unfortunately, the increased demand for these products increases the competition and therefore the price of food products from these sources. Cellulose is one of the most abundant polysaccharide in the nature. Its sources include agricultural and forestry residues, portion of municipal solid waste, and herbaceous and woody crops (Lynd et al. 1991). Bioethanol from cellulose, is therefore a very promising avenue of biofuel as it does not compete with food products (Kemppainen and Shonnard 2005; Sticklen 2008). Producing ethanol from cellulose usually contains two steps. First, the hydrolysis of cellulose to glucose; and second, its fermentation to ethanol. Although the first process can be performed by either chemical or enzymatic process, the later one is preferred because it is cleaner and the process can be controlled by adjusting the reaction conditions easily (such as pH, temperature, etc.) (Mabee and Saddler 2010).

Cellulase refers to a class of enzymes which can catalyze the hydrolysis of cellulose to glucose. It is usually composed of three enzymes: endoglucanase (EC 3.2.1.4), cellobiohydrolase (EC 3.2.1.91) and cellobiase (EC 3.2.1.21) (Percival Zhang et al. 2006). Endoglucanase and cellobiohydrolase are responsible for decreasing degree of polymerization of cellulose to produce cellobiose, which are further hydrolyzed to glucose by cellobiase (Ortega et al. 2001). Unfortunately, difficulty of separation and recovery of free cellulase from the solution after the hydrolysis process limits the reusability of the enzyme which highly precludes the scales of this application because of the high cost of the enzyme (Cerveró et al. 2010). Enzyme immobilization is one effective way, which allow enzyme to reuse, and therefore, reduce the cost of bioethanol production (Sheldon 2007).

The immobilization methods and immobilized carriers are two important factors that significantly influence the properties of biocatalysts. Generally, the immobilization methods can be classified into physical adsorption and covalent binding (Chibata 1978). Physical adsorption is one of the earliest immobilization methods reported in the literature, which can be further categorized into adsorption (by electrical binding, hydrogen binding, and hydrophobic adsorption), entrapment (inside polymer matrix) (Cass and Ligler 1999). It is still widely used, due to its simple and economical process, and limited loss of enzymatic activity (Zhang et al. 2015). The major advantage of physical adsorption is high retention of enzymatic activity. Zhang et al. (2015). demonstrated that cellulase immobilized on modified Fe_3_O_4_ magnetic nanosphere by electrostatic binding can retain 87 % native activity. Mubarak et al. (2014) reported that the specific activity of the immobilized cellulase on functionalized multiwall carbon nanotubes by hydrogen binding was even higher than that of free cellulase. This high retention of immobilized cellulase is reportedly due to the weak interaction between carriers and cellulase molecules, which minimize the change of conformational structure and active center of cellulase molecules (Cass and Ligler 1999). However, this weak interaction also causes enzyme desorption and poor reusability of immobilized cellulase, which are the major disadvantages of physical adsorption (Cass and Ligler 1999). Mishra and Sardar (2015) reported that, with 30 min incubation in CMC for each cycle, the immobilized cellulase on nano-silver and gold can be reused 6 times with 73–78 % initial activity retained. The retained activities of the immobilized cellulase on functionalized multiwall carbon nanotubes by Mubarak et al. (2014) were 52 % for the 6th recycle and 26 % for the 8th recycle. The majority of literature for physical absorption immobilization of enzymes do not report reusability (Takimoto et al. 2008; Chang et al. 2011; Hartono et al. 2010; Zhang et al. 2015; Tebeka et al. 2009; Safari Sinegani et al. 2005).

Covalent bonding is the second main method of enzyme immobilization. The main drawback of this method is major loss of immobilized enzyme activity, due to the stable nature of the covalent bounds between the carriers and the cellulase molecules. The decrease in activity is likely due to changes in the conformational structure of cellulase molecules and decrease in the degree of movement of the cellulase molecules (Cass and Ligler 1999). The typical specific activity of immobilized cellulase by covalent binding is below 52 % (Li et al. 2007, 2013; Wang et al. 2015). However, the stable covalent binding also leads to very high reusability of immobilized cellulase (Wang et al. 2015; Li et al. 2013; Qi et al. 2015; Zang et al. 2014). This major advantage make covalent binding very promising in industrial applications.

Selection of immobilized carriers plays an important role in enzyme immobilization. Preferred characteristics of an enzyme carrier include chemical stability, physical strength, and cost effectiveness (Brena and Batista-Viera 2006). Researchers applied many kinds of materials as carriers for immobilization of cellulase, such as nano-silver and gold particle (Mishra and Sardar 2015), functionalized multiwall carbon nanotubes (Mubarak et al. 2014), copolymers (Tata et al. 2015), silicate clay minerals (Safari Sinegani et al. 2005), modified Chitosan Beads (Dinçer and Telefoncu 2007), modified activated carbon (Anuradha Jabasingh and Valli Nachiyar 2011). Recently, Fe_3_O_4_ magnetic nanoparticle have been paid much attention by researchers for immobilization of cellulase, due to its easy separation from the hydrolysis solution when applying magnetic field (Xu et al. 2011; Zhang et al. 2015; Li et al. 2013; Qi et al. 2015; Zang et al. 2014). Unfortunately, the preferred pH condition for immobilized cellulase is slightly acidic (5) dissolving the Fe_3_O_4_ back to Fe^2+^ and Fe^3+^. Also, the aggregation of Fe_3_O_4_ magnetic nanoparticle was commonly found in research, which decreases the mass transfer rate in solution (Zang et al. 2014, 2015). Silica gel has been widely used in immobilization of enzymes (Han et al. 2005; Díaz and Balkus 1996; Yiu et al. 2001; Yiu and Wright 2005; Lei et al. 2006; Qiao et al. 2005, 2006; Budi Hartono et al. 2009). Its advantages of low cost, chemical stable in acid environment, good dispersion in solution and large surface area make it more favorable than Fe_3_O_4_ magnetic nanoparticle for immobilization of cellulase. Physical adsorption has been applied for immobilization of cellulase on silica gel (Takimoto et al. 2008; Chang et al. 2011; Hartono et al. 2010; Dragomirescu et al. 2012; Ungurean et al. 2013). This immobilization technique utilizes the porous property of silica gel, and entraps cellulase molecules into pores. Takimoto et al. (2008) varied the pore size of silica gel to achieve to highest specific activity of the immobilized cellulase. Hartono et al. (2010) modified silica gel with different kinds of organosilanes to increase the electrical charge of the surface or the hydrophobic affinity of the surface, and therefore increase the immobilized cellulase loading and activity. Others like Dragomirescu et al. (2012) and Ungurean et al. (2013) entrapped the cellulase molecules into sol–gel matrix. However, because of the weak carrier-molecule interaction, which is the inherent defect of physical adsorption, reusability of the immobilized cellulase was rarely reported.

The application of covalent bonding enzyme immobilization with a silica gel carrier provides an opportunity of significantly improving the reusability of a carrier that is low cost, a density closer to the slurry mixture and high surface area. Hartono et al. (2010) modified silica gel with 3-APTES to form amino group terminated surface for immobilization of cellulose. Amide bonds were formed between carboxylic acid functional groups of cellulase molecules and amino group terminated surface. However, the carboxylic acid functional groups come from the aspartic and glutamate acid residues from cellulase are the active sites responsible for hydrolysis of cellulose. Huge loss of the specific activity of immobilized cellulase were observed by Hartono’s (2010) group.Therefore, a new immobilization scheme applying the covalent binding need to avoid the active sites of cellulase. Glutaraldehyde was widely used as a crosslinker for immobilization of enzymes (Sheldon 2011). It aims the α-amino groups of cellulase, which are not the active sites for hydrolysis of cellulose (Tata et al. 2015). Few paper has reported on immobilization of cellulase on silica gel by covalent binding with glutaraldehyde as a crosslinker. In this present work, silica gel with active primary amino group was formed after pretreatment of 3-APTES (Hartono et al. 2010; Alahakoon et al. 2012). Glutaraldehyde was used as a crosslinker for changing the binding functional group of cellulase. It also acts as a spacer molecule between the carriers and the enzyme to prevent the steric constraints (Tata et al. 2015). Because the final goal of cellulase immobilization is to recycle the enzyme to reduce the cost, reusability is the key factor for immobilization. However, most of the prior studies investigate and optimize the hydrolysis conditions (such as pH, temperature) using immobilized cellulase to achieve the highest enzymatic activity. In this paper, the reusability behavior under different hydrolysis conditions was studied. It was found that the reusability of the immobilized cellulase was low at the condition in which the highest activity was reached. Therefore, the optimal working condition was balanced between the enzymatic activity and the reusability of the immobilized cellulase. Also, enzyme desorption from the carriers while hydrolysis of substrates was observed from many enzyme immobilization, and it is one of the reasons that the activity of immobilized enzyme decreases according to cycles (Mubarak et al. 2014). With the strong carrier-molecule covalent binding and the entrapment effect caused by pores of silica gel, it is successfully demonstrated that no cellulase desorption during the hydrolysis of cellulose using the immobilized cellulase on modified silica gel.

## Results and discussion

### Nitrogen adsorption analysis and enzyme loading of modified silica gel

**Fig. 1:**
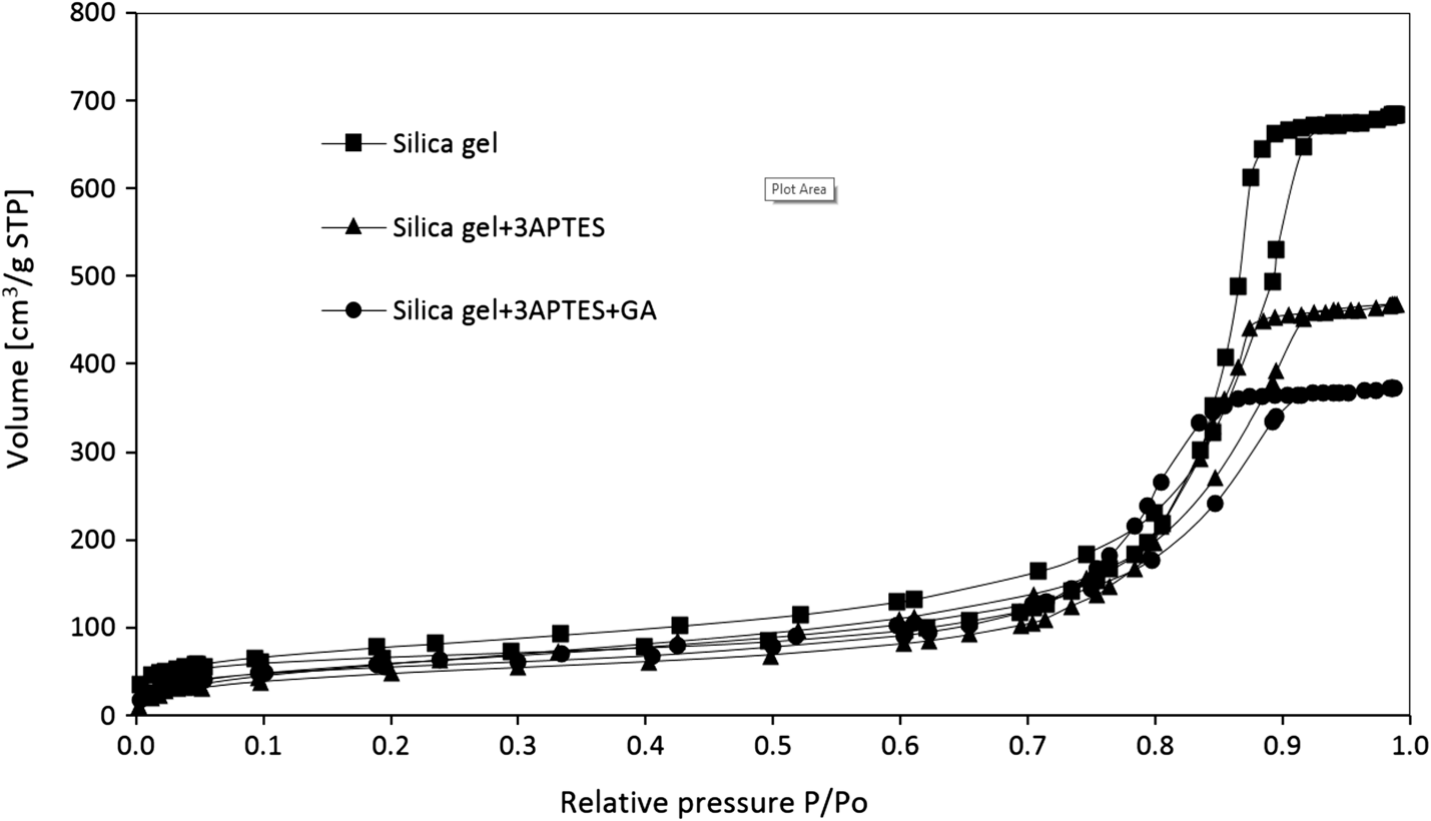
Nitrogen adsorption/desorption isotherms of silica gel, 3-APTES modified silica gel, and glutaraldehyde crosslinked 3-APTES modified silica gel

**Fig. 2:**
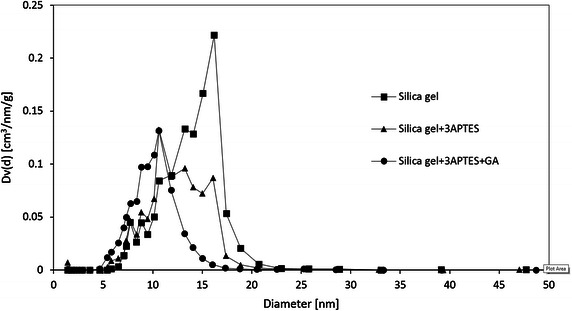
Pore size distribution obtained from nitrogen desorption branch by BJH method

Nitrogen adsorption/desorption analysis was used to characterize the porous structure of immobilization carrier. Figure 1 shows the nitrogen adsorption/desorption isotherms for silica gel, 3-APTES modified silica gel, and glutaraldehyde crosslinked 3-APTES modified silica gel. According to IUPAC classification, they can be classified as type IV isotherms with H_2_ hysteresis loops, which are characteristic of mesoporous materials with a cage-like structure (Matos et al. 2003). Figure 2 shows the pore size distributions which are calculated from nitrogen desorption branch by Barrett–Joyner–Halenda (BJH) method. It can be seen that the pore size of silica gel decreases from 10.6–16.2 to 7.7–10.6 nm after 3-APTES and glutaraldehyde pretreatment. Table 1 shows the summary of pore volume and surface area. The pore volume of pretreated silica gel was 0.6 cm^3^/g, calculated by BJH method. The (Brunauer–Emmett–Teller) BET surface area of pretreated silica gel was 175.5 m^2^/g. The immobilized cellulase on glutaraldehyde crosslinked 3-APTES modified silica gel was 18.8 mg protein (cellulase)/g silica gel.

**Table 1:** Properties of silica gel after immobilization steps

	BET surface area (m^2^/g)	Pore volume (cm^3^/g)
Silica gel	251.9	1.15
Silica gel + 3APTES	159.9	0.78
Silica gel + 3APTES + GA	175.5	0.60

### Characterization of immobilized cellulase

**Fig. 3:**
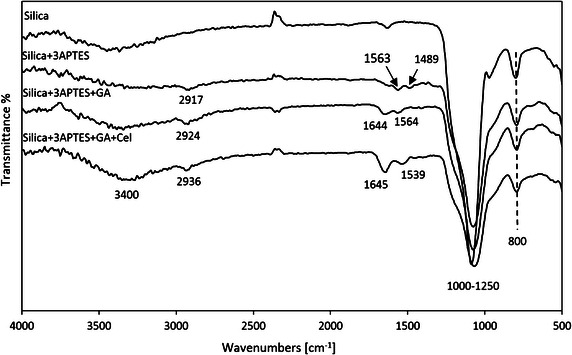
FTIR spectra of silica gel, 3-APTES modified silica gel, 3-APTES modified glutaraldehyde crosslinked silica gel, and immobilized cellulase

The Fourier Transform Infrared (FTIR) spectra of silica gel, 3-APTES modified silica gel, 3-APTES modified glutaraldehyde crosslinked silica gel, and cellulase immobilized silica gel are given in Fig. 3. For silica gel, the Si–O–Si asymmetric stretching vibration at 1000–1250 cm^−1^, OH bending vibration at 800 cm^−1^ appeared (Vansant et al. 1995; Michau and Barboiu 2009). After 3-APTES modification, a new band at 2917 cm^−1^ represented C-H stretching vibration (Vansant et al. 1995). The new peaks at 1563 and 1489 cm^−1^ were attributed to the formation of an aminebicarbonate salt [–NH_3_^+^(HCO_3_)^−^], which was because of drying 3-APTES modified silica gel in room environment (Vansant et al. 1995). The new peak at 1644 cm^−1^ appeared after glutaraldehyde crosslinking suggested imine bond (C=N) vibration, which was formed between glutaraldehyde and 3-APTES layer (Saini et al. 1993; Marin et al. 2012). After cellulase immobilization, the characteristic bands of protein at 1645 and 1539 cm^−1^ associated with C=N vibration at 1644 cm^−1^ appeared in the spectrum (Zang et al. 2014). The broad band at 3400 cm^−1^ after immobilization of cellulase was due to association intermolecular bonds from O–H stretching vibration with N–H stretching vibration in the cellulase molecules, which confirmed successfully immobilization of cellulase onto pretreated silica gel (Rangnekar et al. 2007).

### Modification of immobilization steps

In the immobilization process, the factors that affected the activity of the immobilized cellulase included the saturation and the thickness of the 3-APTES layer. 3-APTES exhibited a fast adsorption on silica gel surface. The monolayer of 3-APTES reached equilibrium within minutes on the silica gel surface (Vandenberg et al. 1991). Organic solvent (toluene) was chosen for 3-APTES in order to control further adsorption and the hydrolysis of 3-APTES (Vansant et al. 1995). The activity of the immobilized cellulase was observed no change for 20–24 h’s immobilization of 3-APTES in toluene. Therefore, 24 h was sufficient time for 3-APTES to saturate the silica gel surface.

**Fig. 4:**
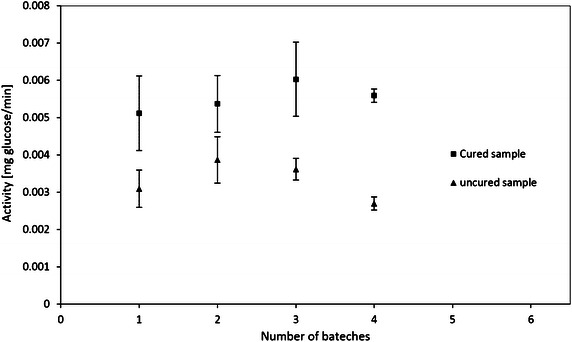
Effect of curing process after 3-APTES modification on the activity of the immobilized cellulase

The curing process after 3-APTES immobilization has a significant impact on the activity of the immobilized cellulase as shown in Fig. 4. The activity of the immobilized cellulase after curing was 1.7 times higher than a similar experiment without curing. The curing process in air environment can cause hydrolysis and possible oligomerization of 3-APTES immobilized. The oligomerization can decrease the thickness of the 3-APTES layer and minimize the changes of pore size of the silica gel (Vandenberg et al. 1991). Therefore curing will increase the cellulase loading on the modified silica gel, and increase the overall activity of the immobilized cellulase.

### Effect of initial concentration of cellulase solution on immobilized cellulase

**Fig. 5:**
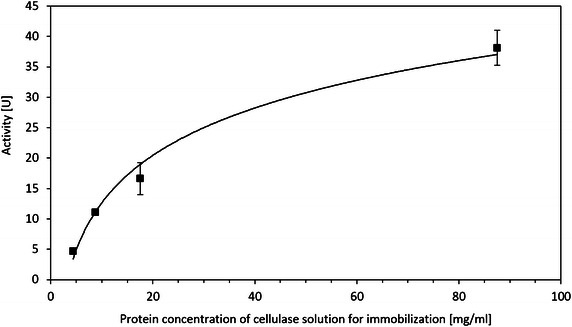
Activity versus protein conc. of cellulase solution for immobilization

**Fig. 6:**
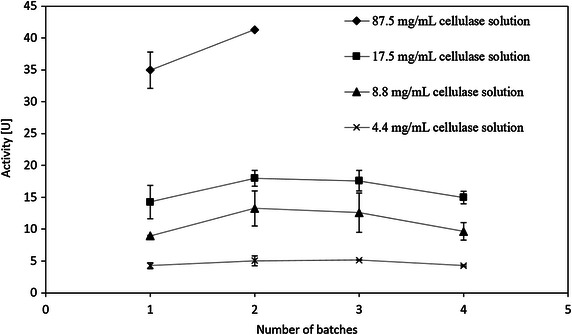
Effect of initial concentration of cellulase solution on the immobilized cellulase

Figure 5 shows the results of enzymatic activity versus the protein concentration of the cellulase solution for immobilization. The non-linear increasing curve trend is due to the Langmuir adsorption isotherm, as well as the mass transfer of produced glucose and CMC. This figure suggests that although the undiluted cellulase solution for immobilization would produce the highest absolute activity, the efficiency of utilization of enzyme is higher at lower dilutions due to losses at the immobilization step (consuming more enzyme) and the hydrolysis step (low mass transfer coefficient caused by cellulase immobilized deep in the pores). Therefore, the optimal dilution of the cellulase solution for immobilization appears to be five. The reusability of the immobilized cellulase by different protein concentrations of cellulase solution is shown in Fig. 6. The activity does not change after 4 batches of hydrolysis of CMC. This indicates that all the cellulase is firmly immobilized on the silica gel surface, and it is independent of the protein concentration of the cellulase solution for immobilization.

### Activity of the immobilized cellulase

Cellulase was immobilized on 3-APTES and glutaraldehyde pretreated silica gel surfaces as described. The activity was determined by testing the produced glucose in 1 % w/v (10 g/L) CMC solution dissolved in 10 ml pH = 6 acetate buffer (50 mmol/L) at 20 °C. One unit of cellulase activity was defined as nmol glucose produced per minute. The activity of immobilized cellulase using 3-APTES and glutaraldehyde pretreatment is 474 ± 20 U per unit gram of immobilized silica gel. The activity of unit enzyme mass of immobilized cellulase is 24 ± 6 U/mg, while the one of the free cellulase is 352 ± 43 U/mg. That is: the specific activity of immobilized cellulase is 7 ± 2 % compared with the similar amount of free cellulase. The denaturation of the immobilized cellulase was due to the decrease in degree of movement of the cellulase molecules after covalent binding, which was commonly found in the studies of immobilization of enzyme (Spahn and Minteer 2008).

### Reusability of the immobilized cellulase

**Fig. 7:**
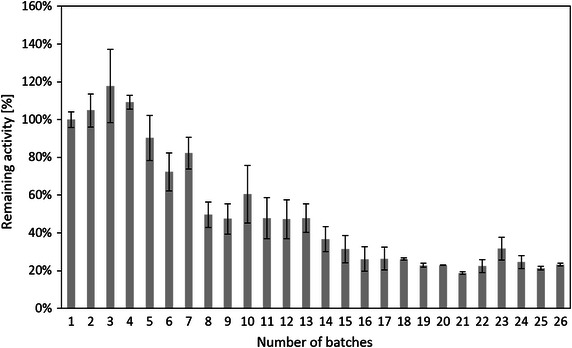
Reusability of immobilized cellulase according to batches

Reusability is an important issue for immobilized cellulase in industrial application (Dinçer and Telefoncu 2007; Alahakoon et al. 2012; Wu et al. 2012). The reusability of immobilized cellulase on modified silica gel was shown in Fig. 7. The relative activity of the immobilized cellulase retained 100–82 % initial activity from 1st to 7th cycle, 60–48 % from 8th to 13th cycle, and 36–23 % from 14th to 26th, respectively. In comparison, the immobilized cellulase was applied to hydrolyze CMC solution 3 h for each cycle, which is longer than previously reported (Mubarak et al. 2014; Mishra and Sardar 2015; Wang et al. 2015; Qi et al. 2015; Anuradha Jabasingh and Valli Nachiyar 2011; Abraham et al. 2014; Gokhale et al. 2013; Verma et al. 2013). The immobilized cellulase exhibits remarkable reusability up to 13 recycles. Table 2 shows the comparison of reusability with other studies. Cellulase entrapped in a MeTMOS/TMOS (3:1 molar ratio) made sol–gel matrix can be reused 6 times with 20 % initial activity retained (Ungurean et al. 2013). Mubarak et al. (2014) reported that cellulase immobilized on acid pretreated MWCNTs by physical adsorption retained 26 % initial activity for the 8th recycle. In another study, 55 % initial activity was retained after 4 recycles when cellulase covalently bound to magnetic graphene nanoparticles (Gokhale et al. 2013). Cellulase immobilized on magnetic nanoparticles via covalent binding can be reused 6 recycles with 40 % initial activity retained (Abraham et al. 2014). The possible reasons of activity loss after each cycle might be immobilized enzyme loss during separation and washing processes after each cycle, enzyme denaturation, and enzyme leak (desorption) (Zang et al. 2014).

**Table 2:** Cellulase immobilization carriers, techniques, and reusability of the current work and other researches

Immobilization carrier	Immobilization technique	Reusability			Refs.
		Times	Time for each cycle	Residual activity (%)	
Sol–gel matrix	Sol–gel entrapment	6	24 h	20	Ungurean et al. (2013)
Sodium alginate gel beads	Sol–gel entrapment and crosslinking	7	N/A	58.37	Wang et al. (2015)
Functionalized multiwall carbon nanotubes	Physical adsorption	8	30 min	26	Mubarak et al. (2014)
Ultrafine Eri silk microparticles	Physical adsorption	8	10 min	50	Verma et al. (2013)
Magnetic porous terpolymers	Covalent binding	6	30 min	48.2	Qi et al. (2015)
Magnetic graphene nanoplatelets	Covalent binding	4	1 h	55	Gokhale et al. (2013)
Magnetic nanoparticles	Covalent binding	6	30 min	40	Abraham et al. (2014)
Modified silica gel	Covalent binding	10	3 h	60	Current work

**Fig. 8:**
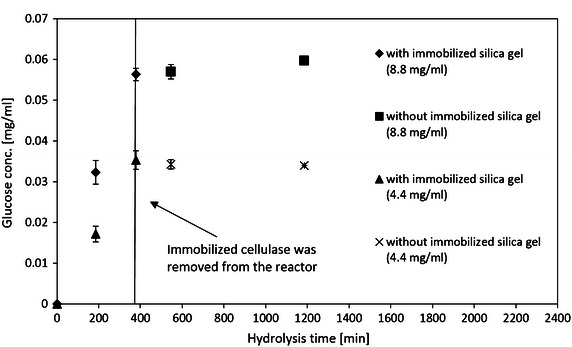
Hydrolysis time versus produced glucose concentrations

Enzyme leak (desorption) is a crucial issue for immobilization of enzyme, and it was observed by many researchers for immobilization of cellulase (Zang et al. 2014; Hartono et al. 2010; Ungurean et al. 2013; Spahn and Minteer 2008). Hartono et al. (2010) found that up to 7 % of immobilized cellulase was desorbed from silica in citrate buffer after 14 day’s storage at 4 °C. Compared with cellulase leak in buffer solution (in washing and storage processes), it is expected that desorption during the hydrolysis of CMC would be more severe, since the binding between immobilized cellulase and CMC provide another driving force to pull the cellulase off the immobilized carrier. The cellulase leak (desorption) in hydrolysis of CMC can make the measured activity and reusability of immobilized cellulase inaccurate (Zang et al. 2014). Because free cellulase is more active than immobilized cellulase, a small amount of cellulase leaked (desorbed) would result in a high enzyme activity. Linear enzyme activity loss of immobilized cellulase was observed according to number of recycles in many prior studies, which suggests leaked (desorbed) cellulase strongly contributed to the activity and the lack of reusability of immobilized cellulase (Mubarak et al. 2014; Wang et al. 2015; Qi et al. 2015; Ungurean et al. 2013). In order to test the enzyme leak (desorption) of immobilized cellulase during hydrolysis of CMC, the immobilized cellulase was centrifuged from the CMC solution after 6 h and then the reactor was kept running. Figure 8 depicts that the immobilized silica gel in 8.8 mg/ml cellulase solution and 4.4 mg/ml cellulase solution, the produced glucose concentration never increased after centrifuging the immobilized silica gel. This strongly indicates that there is no enzyme desorption during the hydrolysis of CMC regardless of the protein concentration of the cellulase solution for immobilization. This was likely due to the entrapment of cellulase molecule in silica gel pore structure and the strong covalent bound between the cellulase molecule and the silica gel surface, which make the specific activity low but maximize the reusability.

**Fig. 9:**
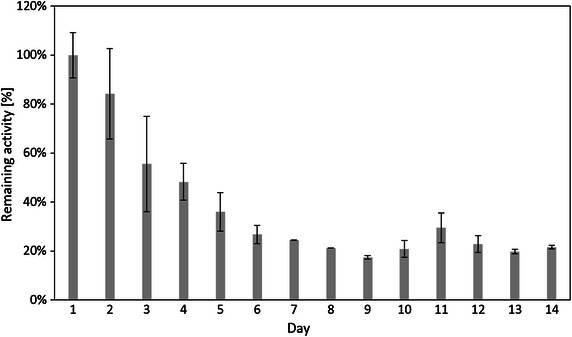
Reusability of immobilized cellulase according to days

Since there was no enzyme leak from the immobilized silica gel, the activity loss could be attributed to denaturation of the immobilized cellulase. The immobilized cellulase exhibited 3 stages of the activity loss in reusability, i.e., the first stage which was from 1st to 7th cycle (the activity retained 100–82 %), the second stage which was from 8th to 13th cycle (the activity retained 60–48 %), and the third stage which was from 14th to 26th cycle (the activity retained 36–23 %). A possible explanation of this behavior is that the immobilized cellulase outside the pores of silica gel was denatured after the first stage, while the one near the pores of silica gel was denatured after the second stage. The porous structure of the silica gel lowered the biological activity of the immobilized cellulase, however it also likely protected the cellulase molecules immobilized in the pores from the conformational structure shifting (Luckarift et al. 2004). Therefore, the activity of immobilized cellulase remained stable after the 13th cycle. This is also confirmed by Fig. 9, which shows that the activity of immobilized cellulase barely decreases after 5th day up to 14th days (from 14th cycle to 26th cycle).

The storage stability of an enzyme is another important factor that limit its applications. The immobilized silica gel was operated first batch and then washed by DI water. After centrifuging and removing supernatant, the deposition (immobilized silica gel) was sealed and kept at 4 °C in the refrigerator for 38 days. The second activity experiment was performed after the 38 day incubation period and the immobilized enzyme on the silica gel still retained 92.4 % of its initial activity.

### Effect of temperature on the immobilized cellulase

**Fig. 10:**
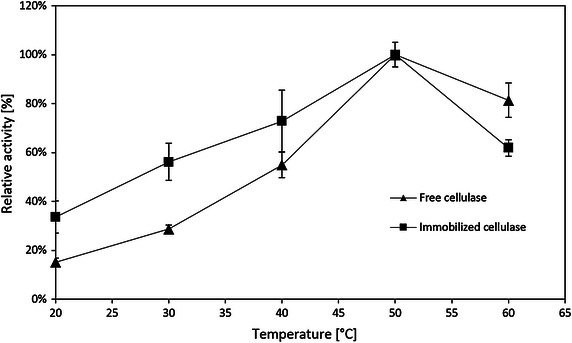
Activity of the immobilized and free cellulase according to temperatures for hydrolysis of CMC

Temperature is an important factor for hydrolysis reaction by enzymes. The activities of the free and immobilized cellulase at different temperatures are shown in Fig. 10. The results show that the activities of both the free and immobilized cellulase have the same trend: the activity increases from 20 to 50 °C and deceases after 50 °C, which is consistent with many other studies (Yu et al. 2012; Li et al. 2015). The immobilized cellulase on modified silica gel exhibited better relative enzymatic activity than free cellulase below 50 °C. Compared with free cellulase, an increase of 18–27 % relative activity was observed for immobilized cellulase from 20 to 40 °C, respectively.

**Fig. 11:**
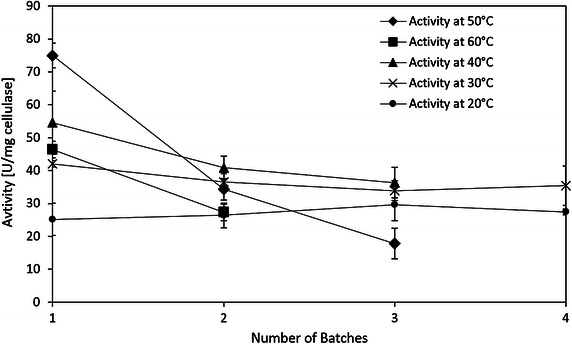
Reusability of the immobilized cellulase at different temperatures

**Fig. 12:**
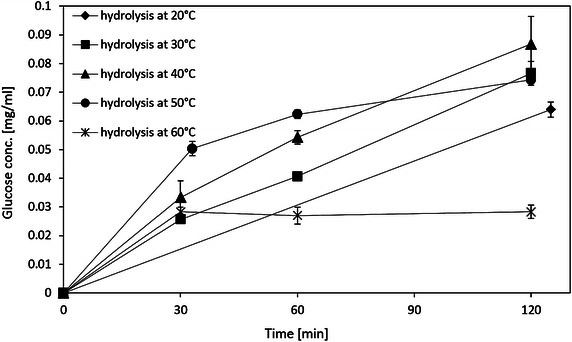
Time-dependent curve of the immobilized cellulase

Reusability of immobilized cellulase was performed at 40, 50, and 60 °C, since the activities of both immobilized cellulase and free cellulase in this range exhibited peak area in Fig. 10. Figure 11 shows that the activities decrease to 24 and 59 % of the initial activities for the third batch at 50 °C and the second batch at 60 °C, respectively. At 40 °C, 66.5 % of the initial activity was retained after 3 recycles. And the specific activity of the immobilized cellulase at 40 °C was larger than the one at 50 and 60 °C after 3 recycles. The possible reason is that the immobilized cellulase at high temperature, i.e., 50 and 60 °C, appears to denature relatively rapidly. Other researchers also observed the same thermal effect (Mao et al. 2006; Wang et al. 2015). Figure 12 clearly shows that the produced glucose concentration linearly increases at 20 to 40 °C for at least 2 h; however, it rapidly reaches its plateau at 50 °C. This behavior is even more obvious at 60 °C, at which the glucose concentration reaches its plateau at 30 min. Therefore, although the initial activity at 50 °C is the highest, 40 °C appears to be the optimal temperature for hydrolysis of CMC using immobilized cellulase.

### Effect of pH on immobilized cellulase

**Fig. 13:**
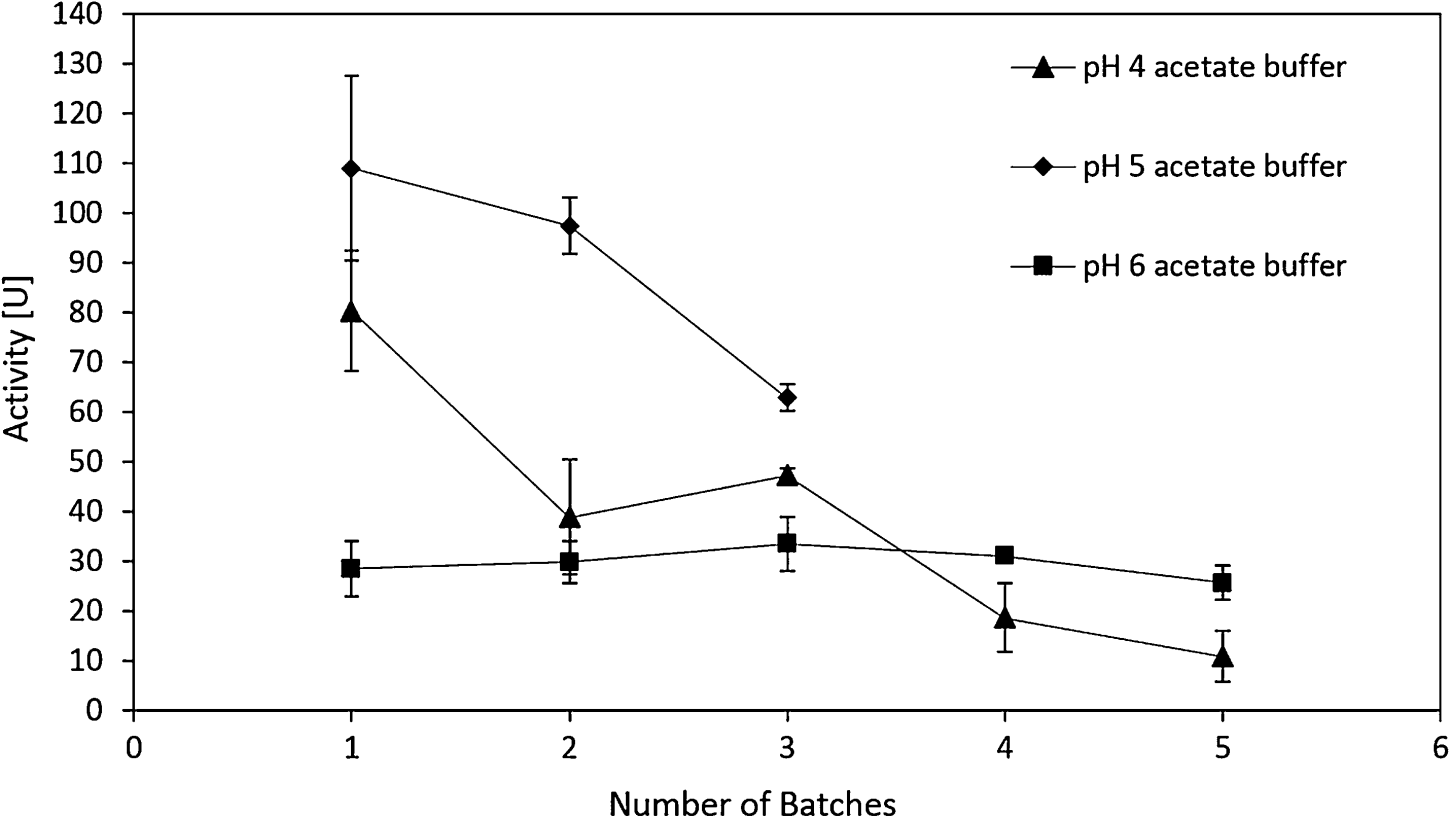
Reusability versus pH for hydrolysis of CMC solution

The pH for hydrolysis of CMC solution was also studied. The 17.5 mg/ml cellulase solution in DI water was used for immobilization. The CMC solution was prepared in pH 4, 5, 6 acetate buffer (50 mmol/L) respectively for hydrolysis at 20 °C. Figure 13 shows the results. The activity of the immobilized cellulase reached highest at pH 5, which was about 4 times higher than the one at pH 6 for the first cycle. However, the reusability at pH 5 was low. The activity of the third cycle rapidly decreased to 58 % compared to the first cycle. The reusability at pH 4 was even worse. Only 14 % activity retained after the fifth batch. The possible reason was that the enzyme rapidly desorbs at pH 4 and 5. Thus, the high activity for the first batch was due to the free cellulase in the CMC solution, since the specific activity of the immobilized cellulase was only 7 % compared to the same amount of the free cellulase. Therefore, considering the reusability, although the cellulase was slightly activated at pH 4 and 5, pH 6 was the optimal pH for hydrolysis of CMC.

## Conclusions

In this study, cellulase was immobilized covalently on silica gel modified by 3-APTES using glutaraldehyde as a cross-linker. Although the specific activity of the immobilized silica gel was 7 ± 2 % of free cellulase, a very high reusability was observed over a period of 2 weeks (up to 26 different batches in 14 days). No enzyme desorption was observed during the hydrolysis of CMC solution at the optimized conditions of pH 6 acetate buffer. The immobilized cellulase exhibited enzymatic activity higher than free cellulase for temperatures below 50 °C. Silica gel was demonstrated to be an excellent substrate for immobilization, due to the close density to the slurry and protective characteristics of the pores. Covalent bonding of the cellulase to the silica gel was necessary for the outstanding reusability observed.

## Methods

### Chemicals

Cellulase (Accellerase 1500 from Danisco US Inc., Genencor Division) was centrifuged and the supernate was used for further testing because the micro-particles in the original solution released proteins which did not have activity but affected the protein assay. Silica-Amorphous precipitated, (3-aminopropyl) triethoxy-silane (3-APTES), toluene (anhydrous, 99.8 %), glutaraldehyde (GA) solution (grade I, 50 %), carboxymethylcellulose sodium salt (CMC), sodium acetate trihydrate, and fluorescamine were purchased from Sigma.

### Pretreatment of silica gel

#### Modification of silica gel

Silica gel involved in this project was modified by 3-APTES to get amino-group terminated surface. 0.06 g silica gel was incubated with 10 % v/v 3-APTES prepared in toluene at 30 °C for 24 h in incubator shaker (Innova*™* 4000, New Brunswick Scientific) with 300 rpm shaking speed. The modified silica gel was followed by a washing step with 1.2 ml toluene for 4–5 times to remove the unbound 3-APTES. Then the modified silica gel was cured on hot plated at 40 °C for 24 h. The modified silica gel is stable for months if sealed (Vansant et al. 1995).

#### Crosslinking modified silica gel

Glutaraldehyde was used as a crosslinker and a space arm between the 3-APTES layer and the cellulase layer. The 3-APTES modified silica gel was immerged into 2 % v/v glutaraldehyde solution which was prepared with pH = 6 acetate buffer (50 mmol/L) for 30 min at room temperature. After this step, an aldehyde terminated silica gel surface was formed. Then modified silica gel was washed by 1.2 ml DI water 4–5 times to remove the unbound glutaraldehyde.

### Immobilization of cellulase

**Fig. 14:**
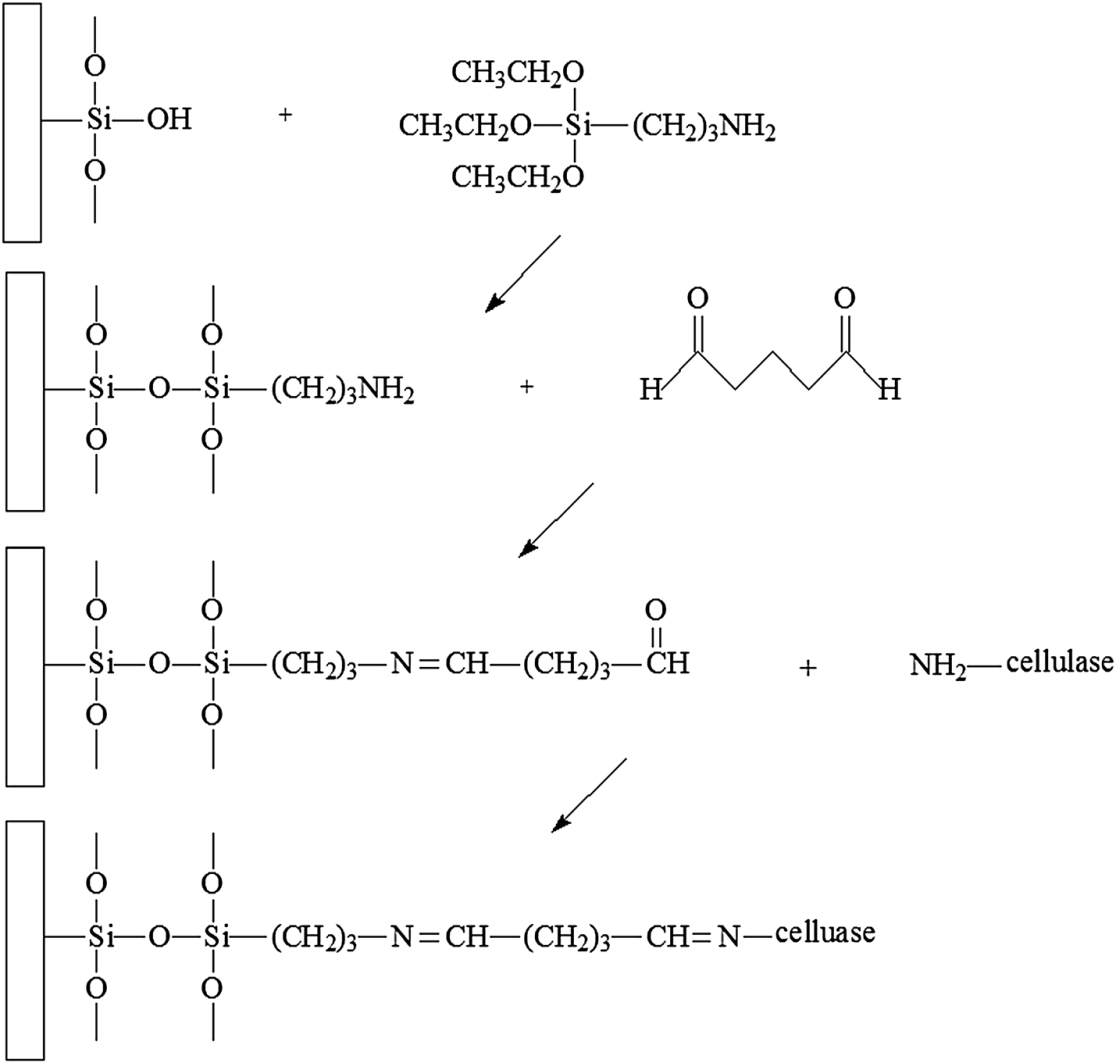
Mechanism of immobilization with 3-APTES

0.06 g modified silica gel was immersed into 1 ml of 5× diluted cellulase with DI water (17.5 mg protein/ml) for 24 h at room temperature in incubator shaker with 300 rpm shaking speed. The incubator shaker utilized for immobilization of 3-APTES and cellulase was to make the silica gel uniformly distributed in the solution and kept a certain temperature. After remove the residue immobilization solution, the immobilized silica gel was washed 5 times with DI water. Only 0.1 % of cellulase was detected in the supernate of the fifth wash suggesting that no further washes were required. Figure 14 shows the mechanism of immobilization with 3-APTES. The cellulase solution after immobilization and the water waste were kept together in a 100 ml volumetric bottle for protein assay in order to determine the amount of immobilized cellulase.

### Characterization methods

The Fourier Transform Infrared Spectroscopy (FTIR) was used for recording the chemical composition of the samples. The samples were prepared by 100 % pure silica, 3-APTES modified silica, 3-APTES modified glutaraldehyde crosslinked silica gel, and cellulase immobilized silica, respectively. The spectra were recorded at room temperature in the 400–4000 cm^−1^ range using Thermo Scientific NICOLET IR100 FT-IR Spectrometer. The pore size and pore volume were calculated by nitrogen adsorption and desorption using the Barrett–Joyner–Halenda (BJH) method using NOVA 2000 High-Speed Surface Area and Pore Size Analyzer. The surface area was calculated by Brunauer–Emmett–Teller (BET) method.

### Protein assay to determine the amount of cellulase

Fluorescamine protein assay was used to determine the amount of immobilized cellulase, in which 5 mg/ml bovine serum albumin (BSA) stock was prepared for a standard curve. Table 3 shows the standard curve. One ml of 5× diluted cellulase solution for immobilization was added into a 100 ml volumetric bottle, and diluted with DI water. Three ml of the diluted solution was used for protein assay. The samples were measured using fluorescence spectrometer at excitation wavelength 390 nm and emission wavelength 460 nm. The amount of immobilized cellulase was calculated by:

$${\text{Mass of immobilized cellulase}} = {\text{C}}_{\text{i}} {\text{V}}_{\text{i}} - {\text{C}}_{\text{f}} {\text{V}}_{\text{f}}$$

where C_i_ is the initial protein concentration, Vi the initial volume of cellulase solution, C_f_ the final protein concentration after immobilization, V_f_ the final volume of cellulase solution after immobilization (water washes included). The maximum percentage error of C_i_ and C_f_ ranged from 3 to 4 %. The magnitude of measured difference of enzyme mass before and after the immobilization step was 11–14 %.

**Table 3:** Standard curve of fluorescamine protein assay

Protein conc. (mg/ml)	BSA stock (ml)	Water (ml)	Fluorecamine (ml)	Incubation (min)
0	0	3.000	0.05	30
0.025	0.015	2.985	0.05	30
0.050	0.030	2.970	0.05	30
0.100	0.060	2.940	0.05	30
0.200	0.120	2.880	0.05	30
0.300	0.180	2.820	0.05	30

### Activity of hydrolysis of CMC by the immobilized and the free cellulase

Activities of the immobilized and free cellulase were determined by hydrolysis of 1 % w/v (10 g/L) CMC solution dissolved in pH = 6 acetate buffer (50 mmol/L). 0.06 g immobilized silica gel was immersed into 10 ml CMC solution and stirred well in the reactor, which maintained temperature of 20 °C in a water bath. Samples were taken after 1, 2, and 3 h, which were centrifuged for 5 min at 10,000 rpm. The supernate was collected, and its glucose concentration was tested by YSI 7100 MBS (Multiparameter Bioanalytical System from YSI Life Sciences).

### Reusability of immobilized cellulase

The reusability of immobilized cellulase was studied by hydrolysis of 10 g/L CMC solution in pH = 6 acetate buffer at 20 °C. The CMC assay, samples taking and testing were similar as described in “Activity of hydrolysis of CMC by the immobilized and the free cellulase” section. For each cycle, the hydrolysis of CMC by immobilized cellulase took 3 h. After each cycle, the immobilized cellulase was centrifuged and washed by DI water. The immobilized cellulase was then collected by centrifuging, and dispersed in fresh CMC solution for the next cycle. The initial concentration of cellulase solution for immobilization of silica gel was 17.5 mg/ml (5× diluted). The first cycle was recognized as the control group, and its activity was defined as a relative activity of 100 % (Mubarak et al. 2014).

In order to study the activity’s decay of immobilized cellulase according to days, 2–3 cycles’ hydrolysis of CMC by immobilized cellulase were performed on each day. The immobilized cellulase was collected and washed after each cycle as that stated above. The immobilized cellulase was washed and stored at 4 °C in refrigerator at night. The daily activity of immobilized cellulase was calculated by averaging the activities of each cycle on that day. The activity of the first day was recognized as the control group, and defined as a relative activity of 100 %.

### Effect of initial concentration of cellulase solution on immobilized cellulase

The activities of immobilized cellulase from different time-diluted cellulase solutions for immobilization were tested. Silica gel was modified by 3-APTES and crosslinked by glutaraldehyde as described in “Pretreatment of silica gel” section. Then the pretreated silica gel was immerged in 20×, 10×, 5× diluted and undiluted cellulase solution, respectively. The protein concentrations of the diluted cellulase solutions for immobilization measured by flourescamine protein assay, were 4.4, 8.8, 17.5, 87.5 mg/ml for 20×, 10×, 5× diluted and undiluted cellulase solution, respectively. The activity of the immobilized cellulase was determined by CMC assay as stated in “Activity of hydrolysis of CMC by the immobilized and the free cellulase” section.

### Effect of temperature on immobilized cellulase

The effect of temperature on both free and immobilized cellulase was examined in 10 g/L CMC solution in pH 6 acetate buffer (50 mmol/L) by altering the reaction temperature to 20, 30, 40, 50, and 60 °C. Methodology of samples taking and testing was similar as “Activity of hydrolysis of CMC by the immobilized and the free cellulase” section. The initial concentration of cellulase solution for immobilization of silica gel was 17.5 mg/ml (5× diluted). The highest activities of free and immobilized cellulase were considered as control groups for each series of experiments, respectively, and defined 100 % relative activity (Qi et al. 2015).

### Effect of pH on immobilized cellulase

The effect of pH for hydrolysis of CMC on immobilized cellulase was examined by altering the acetate buffer to pH 4, 5, 6 in the CMC assay in “Activity of hydrolysis of CMC by the immobilized and the free cellulase” section. The immobilized cellulase was performed as stated in “Pretreatment of silica gel” and “Immobilization of cellulase” sections.

## Abbreviations

3-APTES: (3-aminopropyl) triethoxy-silane
GA: glutaraldehyde
CMC: carboxymethylcellulose sodium salt
FTIR: Fourier Transform Infrared Spectroscopy
BJH: Barrett–Joyner–Halenda
BET: Brunauer–Emmett–Teller
BSA: bovine serum albumin
DI water: deionized water
MBS: Multiparameter Bioanalytical System
MeTMOS: methyltrimethoxysilane
TMOS: tetramethoxysilane
